# Facile Modification of Flexible Electrodes via Laser Transfer

**DOI:** 10.3390/ma15072488

**Published:** 2022-03-28

**Authors:** Florin Andrei, Iulian Boerasu, Mihaela Filipescu, Alexandra Palla-Papavlu

**Affiliations:** Lasers Department, National Institute for Lasers, Plasma and Radiation Physics, Atomistilor 409, 077125 Magurele, Romania; florin.andrei@inflpr.ro (F.A.); iulian.boerasu@inflpr.ro (I.B.); mihaela.filipescu@inflpr.ro (M.F.)

**Keywords:** SnO_2_, Pd-SnO_2_, laser transfer, LIFT, electrodes, acetylacetonates

## Abstract

In this work, we report the modification of commercially available electrochemical electrodes with tin oxide (SnO_2_) and Pd doped SnO_2_ (Pd-SnO_2_) via pulsed laser-induced forward transfer (LIFT). The pulsed light irradiation working as in situ pulsed photo-thermal treatment allows for the transfer of SnO_2_ and Pd-SnO_2_ from UV absorbing metal complex precursors onto flexible, commercially available screen-printed electrodes. The laser transfer conditions are optimized and the material transferred under different conditions is evaluated morphologically and chemically, and its functionality is tested against the detection of copper ions. For example, by applying laser fluences in the range 100–250 mJ/cm^2^, the shape and the size of the transferred features ranges from nano-polyhedrons to near corner-grown cubic Pd-SnO_2_ or near cubic Pd-SnO_2_. In addition, the EDX analysis is consistent with the XPS findings, i.e., following laser transfer, Pd amounts lower than 0.5% are present in the Pd-SnO_2_ pixels. First sensing tests were carried out and the transferred Pd-SnO_2_ proved to enhance the cathodic peak when exposed to Cu(II) ions. This photo-initiated fabrication technology opens a promising way for the low-cost and high-throughput manufacturing of metal oxides as well as for electrodes for heavy metal ion detection.

## 1. Introduction

Metal acetylacetonates are compounds derived from the acetylacetonate anion and metal ions [1] and they are currently utilized in a plethora of applications, such as precursors for catalysis [2], for the development of hybrid nanomaterials or membranes with enhanced gas separation performance [3], in energy storage applications [4,5], in sensor designs [6,7], in oxygen reduction reaction [8,9] or in generation of hydrogen [10]. 

Recently, numerous studies dedicated to the film-forming capacity of metal acetylacetonates have been reported, as these films are particularly attractive in optoelectronic applications [11,12] or as corrosion resistant coatings [13]. In addition, films of ZrO_2_, HfO_2_, SnO_2_, etc., prepared from metal acetylacetonate solutions, are particularly appealing due to the low deposition temperatures which make them suitable for being applied to flexible substrates and with a good potential for scaling up [14,15,16]. 

Although there are numerous deposition methods for metal oxide thin films from metal acetylacetonates, such as metal organic chemical vapor deposition technique [17], metal organic coating photolysis process [18], combined reactive magnetron sputtering and plasma decomposition [19], the laser-based methods have been proven as excellent alternatives.

Laser-induced forward transfer (LIFT) is a simple process, where a pulsed laser beam is focused through a transparent substrate (i.e., donor substrate) onto a thin layer (donor layer) [20,21,22,23]. Each laser pulse allows the precise and well localized transfer of the donor layer as a pixel (with different shapes, areas and thicknesses) on a rigid or flexible substrate (receiver substrate) that is placed parallel, in contact to or at short distance from the donor. One of the advantages of LIFT is that it allows the transfer of materials in different phases (liquid [20,24,25,26] or solid [27,28,29,30,31]) with high resolution and without contamination, even if the process is carried out in air. This technique can be successfully applied for processing of sensors [31], organic light emitting diodes [32], flip-chip devices [33], etc. Recently, LIFT was used as method of bactericidal treatment of pathogenic bacterial biofilms in vitro [34]. In the work of Soulis et al. [35], a biosensor for the detection of organophosphorous and carbamate pesticides has been reported, which is based on LIFT of different liquid mixtures, i.e., chitosan, acetylcholinesterase, carbon black applied to modify the surface of the SPE electrodes. 

Furthermore, in [36,37], an approach based on laser-induced forward transfer, i.e., reactive LIFT (rLIFT), has been used to transfer SnO_2_ pixels from SnCl_2_(acac)_2_ precursors. The main advantage of the rLIFT method is related to the low decomposition temperatures of the acetylacetonates combined with the spatial resolution of the LIFT technique. Later on, in a study conducted by A. Palla-Papavlu et al. [38], the rLIFT technique was used for the integration of SnO_2_ and Pd-SnO_2_ in micro-sensors. Starting from a metal precursor, the rLIFT allows the conversion into the corresponding metal oxide as a result of the photochemical and thermal processes. This can be considered as an important advantage as the fabrication process is shorted. It was proven that the rLIFT fabricated sensors based on tin oxide had up to 4 times higher sensitivities than the commercial sensors (with SnO_2_ inkjet printed). In addition, the sensitivity to CH_4_ of the sensors based on Pd-SnO_2_ is significantly increased in comparison with the pure SnO_2_ sensors.

Therefore, due to our continued interest in the development of sensors by applying LIFT and modifications of LIFT, in this work, we have transferred different SnO_2_ and Pd-SnO_2_ pixels onto flexible and low-cost electrochemical sensors and tested them for their ability to detect Cu ions. We have chosen Pd-SnO_2_ due to the fact that noble metal additives are frequently used to improve the performance of metal oxide gas sensors [39]. The novelty of the paper is twofold: on one hand, we focus on the promotion of LIFT as a viable method to decorate electrodes for sensors, and, on the other hand, we aim at improving and developing new flexible sensors by a low-cost, solvent free, environmentally friendly method.

## 2. Materials and Methods

### 2.1. Preparation of the Donor Substrates

SnCl_2_(acac)_2_ (from Merck KGaA, Darmstadt, Germany) is synthesized according to the procedure described in [36,37,38]. SnCl_2_ is mixed with acetylacetone and HCl. One wt% Triton X-100 (from Merck KGaA, Darmstadt, Germany) is added to the final solution in order to improve the wettability when spin coating on the quartz substrates. The solution is filtered with a 1 µm filter and spin coated at 2500 rpm, resulting in thin films with a thickness of approximately 900 nm ± 70 nm. 

A commercially available Pd metal precursor solution (i.e., Pd(acac)_2_) is purchased from Merck KGaA, Darmstadt, Germany and 0.5 wt% of Pd(acac)_2_ is added to the SnCl_2_(acac)_2_ solution applied for spin coating. 

### 2.2. Electrode Modification via Laser-Induced Forward Transfer

Flexible and low-cost SPPEDOTEs (Dropsense ref. P10, Asturias, Spain) with carbon counter electrode (CE), reference electrode (RE) made of silver and working electrode (WE) based on [poly(3,4 ethylenedioxythiophene)] (PEDOT) are used as receiver substrates. The WE consisting of 4 mm diameter PEDOT disk of identical commercial screen-printed electrode (SPE) is modified with SnO_2_ and Pd-SnO_2_ using an in-house laser-induced forward transfer (LIFT) system.

**Figure 1:**
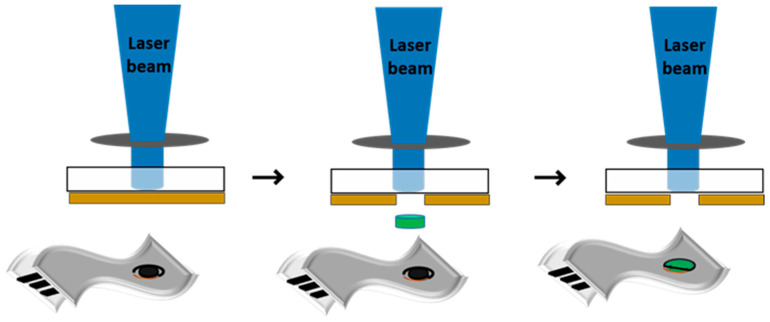
Scheme of the LIFT process for printing SnO_2_ and Pd-SnO_2_ pixels from SnCl_2_(acac)_2_ and Pd-SnO_2_ donors onto flexible electrochemical electrodes.

The LIFT setup (the sketch is shown in Figure 1) used in this work consists of a pulsed ArF laser (193 nm emission wavelength, 20 ns pulse length, 1 Hz repetition rate, from Coherent Inc., Santa Clara, CA, USA) which is guided and imaged with an optical system at the quartz substrate–SnCl_2_(acac)_2_ layer interface and as a result of the rapid increase in pressure at the quartz-SnCl_2_(acac)_2_ interface, a part of the donor layer is transferred (further named pixel) onto the receiving substrate. A computer-controlled xyz translation stage allows the displacement of both donor and receiving substrates with respect to the laser beam. The donor and receiver are placed parallel and in close proximity (<10 µm). All experiments are carried out under ambient pressure at temperatures close to room temperature. The laser fluence is varied over a broad range, i.e., from 100 to 250 mJ/cm^2^. 

### 2.3. Investigation of Electrode Functionality

All chemicals are analytical grade and are used with no further purification. Anhydrous copper sulphate (CuSO_4_; purity ≥ 99.99%) is purchased from Merck KGaA, Darmstadt, Germany. For the preparation of acetate buffer solution, glacial acetic acid and sodium acetate (both from Merck KGaA Darmstadt, Germany) are acquired. 

The electrochemical investigations are carried out with an AutoLab PGSTAT302N (Utrecht, The Netherlands) controlled by NOVA version 1.11 software (Utrecht, The Netherlands). The connection between the potentiostat and electrodes is made using a standard cable connector for screen-printed electrodes. All the sensors are tested for copper detection by cyclic voltammetry measurements scanning the potential from −1.4 V to 0.4 V and reversely using a scan rate of 100 mV/s. An amount of 100 ppm of Cu^2+^ is added to 0.1 M acetate buffer solution (pH = 5.0 upH) and is energetically stirred with for 20 min. 

### 2.4. Surface Investigation of the Transferred Material and Functionalized Electrodes 

The transferred SnO_2_ and Pd-SnO_2_ pixels as well as the donor films prior to ablation are investigated by optical microscopy. The images are acquired with an Olympus SZH 10 Research Stereo microscope (Olympus CO (Europa) GMBH, Hamburg, Germany) coupled with a Stingray F145C CCD camera (Stadtroda, Germany).

Atomic force microscopy (AFM) (XE 100 from Park System, Suwon, Korea) measurements are carried out to analyze the surface roughness of the spin coated SnCl_2_(acac)_2_ and Pd-SnCl_2_(acac)_2_ donor films and SnO_2_, respectively, Pd-SnO_2_ transferred pixels on different areas and dimensions (40 × 40 μm^2^ and 5 × 5 μm^2^). All AFM images are obtained at ambient conditions. Commercial silicon cantilevers are used (OMCL-AC240TS, Olympus cantilevers) with 70 kHz nominal resonance frequency and 2 N/m nominal force constant. 

The surface morphology and chemical composition of both SnCl_2_(acac)_2_ donors and transferred pixels features are examined by scanning electron microscopy (SEM) and energy-dispersive spectroscopy (EDS). These investigations are achieved by using an Apreo FEG High-Resolution Scanning Electron Microscope (HR-SEM), model S LoVac (Thermo Fisher Scientific Inc., Hillsboro, OR, USA) equipped with a Trinity detector system and coupled with an EDAX Trident (EDS-EBSD-WDS) Analysis System (AMETEK Inc., Mahwah, NJ, USA). To assist with simultaneous surface imaging and composition analysis, the involved Apreo S LoVac HR-SEM is operated in High-Vacuum mode at the analytical working distance (10 mm) with 10 kV voltage electron beams and 25 pA beam currents. To completely avoid the charge mitigation issue, a thin layer of gold (Au) (5 nm thick) is sputtered on the samples.

The chemical states of Pd and Sn in the transferred pixels are analyzed by x-ray photoelectron spectroscopy (XPS). The XPS spectra are recorded using a ESCALAB Xi+ system from Thermo Fisher Scientific (Waltham, MA, USA). XPS survey spectra and high-resolution XPS scan spectra are acquired for the SnO_2_ and Pd-SnO_2_ pixels. The survey scans are acquired using Al Kα gun, with spot size 900 μm, pass energy of 50.0 eV, energy step size 1.00 eV, and five scans are accumulated, while for the high-resolution XPS spectra, the pass energy is set to 20.0 eV, and energy step size is 0.10 eV and 35 scans are accumulated. The pressure during the acquisition of the spectra is 5 × 10^−9^ mbar.

Contact-angle measurements are carried out with a KSV CAM101 microscope (from KSV Instruments Ltd., Helsinki, Finland) equipped with a video camera. All contact-angle measurements are obtained by applying the sessile drop method, i.e., a syringe is used which disperses double-distilled water droplets with a volume of 1 ± 0.1 μL. Five different points are measured for every pixel deposited onto the commercial electrodes, and the contact angle reported is the average of these measurements.

## 3. Results and Discussion

### 3.1. Morphological Investigation 

The aim of this study is to get insight into the possibility to use SnO_2_ and Pd-SnO_2_ pixels printed from metal acetylacetonates for the development of flexible electrodes which can detect Cu(II) ions. Therefore, a systematic investigation of the surface of SnO_2_ and Pd-SnO_2_ printed pixels is carried out first.

**Figure 2:**
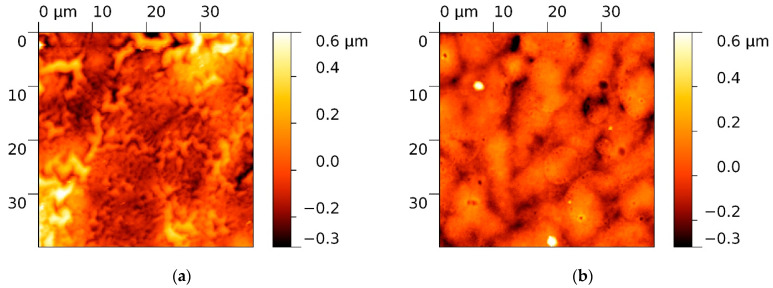
AFM images of the (**a**) SnCl_2_(acac)_2_ donor surface, (**b**) Pd-SnCl_2_(acac)_2_ donor surface.

In order to print the SnO_2_ and Pd-SnO_2_ pixels, reproducible donor films from SnCl_2_(acac)_2_ and Pd(acac)_2_ precursors are fabricated. Two types of donors based on SnCl_2_(acac)_2_ are fabricated, which have distinctive morphologies: (i) “wrinkle”-like structures with predominant valleys (Rsk = −1.105) that induce a high roughness (Rq = 156.17 nm) of the SnCl_2_(acac)_2_ thin layer (Figure 2a) and (ii) a relatively flat surface with an almost symmetrical height distribution (as indicated by the Rsk value close to zero), with lower roughness (Rq = 97.25 nm) and with valleys, as revealed by the negative value of Rsk (−0.379) that characterize the Pd-SnCl_2_(acac)_2_ donor surface. On a large area (40 µm × 40 µm), the surface of this donor has pores with different sizes (0.3–2.5 µm) (Figure 2b).

The parameters describing the surface morphology (Rq, Min, Max, Rsk), as extracted from AFM scans, are collected in Table 1. The average squared roughness value (Rq) represents the standard deviation of the height value in the selected region, Min is the minimum height value of the scanned region and Max is the maximum height value of the region. Another important parameter that can be extracted from AFM investigation is the skewness (Rsk). Skewness is used to measure the profile symmetry about mean line. When the height distribution is symmetrical, Rsk is zero. If the height distribution is asymmetrical, and the surface has more peaks than valleys, the skewness moment is positive, and if the surface is more planar and valleys are predominant, the Rsk is negative. 

**Figure 3:**
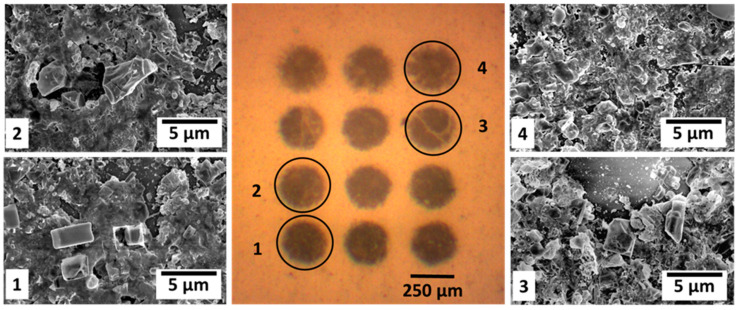
(Center image) Optical microscopy image of a Pd-SnO_2_ pixel array obtained by varying the laser fluence between 100 mJ/cm^2^ (lowest row) and 250 mJ/cm^2^ (top row). Images 1–4 represent scanning electron microscopy images taken in the middle of the Pd-SnO_2_ pixel transferred at: image 1—100 mJ/cm^2^; image 2—150 mJ/cm^2^; image 3—200 mJ/cm^2^; and image 4—250 mJ/cm^2^.

Once reproducible donors are obtained, laser-induced forward transfer is applied to transfer SnO_2_ and Pd-SnO_2_ pixels onto glass substrates. The fluence is varied over a broad range, i.e., from conditions insufficient to break the donor layer to high irradiation fluences (250 mJ/cm^2^), in order to optimize the shape of the transferred pixels. The threshold fluence for the complete removal and transfer of either SnO_2_ or Pd-SnO_2_ thin films from the irradiated area (250 µm in diameter spot) is 100 mJ/cm^2^. All transferred pixels have a circular shape, are regularly arranged, and in some cases, the edges are less uniform, e.g., the pixels at 200 and 250 mJ/cm^2^ have a negligible amount of material scattered outside the pixel area. We found that the transfer of Pd-SnO_2_ pixels occurs for the same conditions as for SnO_2_. An example of optical micrographs of the Pd-SnO_2_ pixels obtained immediately after transfer at different laser fluences are displayed in Figure 3.

An in-depth investigation of the topography is carried out by SEM, which reveals that the distribution of material inside the pixel area is dependent on the laser fluence. The shape and size of the features observed inside the pixel range from nano-polyhedrons, to near corner-grown cubic Pd-SnO_2_, or near cubic Pd-SnO_2_. The high magnification micrographs recorded on the selected pixels, marked 1–4 in Figure 3, reveal the surface morphology and topography of the transferred layer, which was originally the bottom part of the Pd-SnCl_2_(acac)_2_ donor target. Although the transferred material covers the substrate, there are distinguishable visible voids on the pixels’ surface. Noticeably, the number, shape and size of the distinguished voids varies from the pixel marked 1 to pixel 4. While the surface of pixel 1 appears to be coarse and includes a large amount of micro-porosity, the surface of pixel 2 is characterized by a smooth topography and visible macro-porosities. Furthermore, large size cavities are visible on the surfaces of pixel 3 and 4. Some crystalline aggregates are visible on the surface of the transferred layer, as shown in Figure 3. Interestingly, both the density and size of the observed crystals seems to gradually increase from pixel 1 to pixel 4. Another finding in Figure 3 (pixel 3 & 4) is the coincidence of the cavity’s shape and size with those of the observed crystals. According to this observation, it seems that the observed large cavities on the pixel 3 & 4 surfaces (dashed arrow) are formed by crystals pulled out (solid arrow) during the LIFT process.

**Figure 4:**
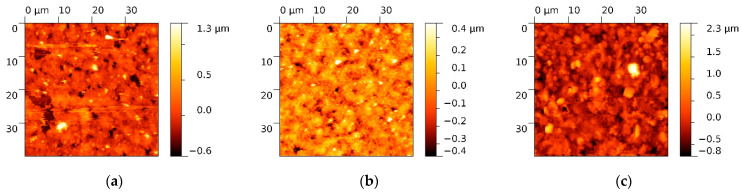
AFM images of (**a**) clean surface of the working electrode, (**b**) SnO_2_ pixel transferred at 100 mJ/cm^2^ laser fluence on the surface of the working electrode, and (**c**) Pd-SnO_2_ pixel transferred at 100 mJ/cm^2^ laser fluence on the surface of the working electrode.

Furthermore, SnO_2_ and Pd-SnO_2_ pixels have been transferred at 100 mJ/cm^2^ laser fluence onto the commercially carbon electrodes in order to further test their functionality by typical cyclic voltammetry. An example of a transferred SnO_2_ and Pd-SnO_2_ pixel onto the surface of the working electrode is shown in Figure 4. Both the SnO_2_ and Pd-SnO_2_ transferred pixel morphology presents irregular grains with micrometric sizes (1–1.5 µm) and large agglomerations (>5 µm). The scanned surface is relatively planar with an almost symmetrical height distribution (Rsk value close to zero), with high roughness (Rq = 412 nm) and valleys (revealed by the negative value of Rsk).

The differences between the surface morphology of the donor layers (i.e., SnCl_2_(acac)_2_ and Pd-SnCl_2_(acac)_2_) and the surface morphology of the transferred material appear due to the transformation of the metal precursor to SnO_2_ and Pd-SnO_2_ during laser transfer as a result of the photochemical and thermal processes. 

### 3.2. Chemical Investigation

The scope of this study is to demonstrate the fabrication of a proof-of-concept system where commercially available electrodes are decorated with thin film pixels by rLIFT technique. In this section, we aim at describing the chemical states of Pd and Sn from the transferred SnO_2_ and Pd-SnO_2_ pixels in order to provide more chemical information for improving the sensitivity of the sensor.

**Figure 5:**
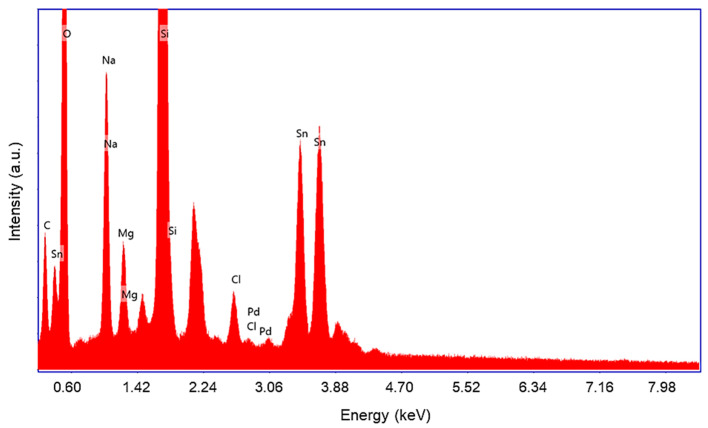
Typical EDX spectra recorded on the transferred Pd-SnO_2_ pixels.

First, in order to assess any modification in the elemental composition by the transfer process, a set of EDX analyses are carried out on the selected pixels from Figure 3. The EDX spectra recorded on pixel 1, which is similar, in terms of detected elements, to the spectra belonging to pixels 2–4, is shown in Figure 5.

The EDX analysis reveals that the transferred material consists of O, Sn and Pd elements. However, there were also detected Na, K, Mg and Cl elements as originated from the target donor synthesis process. The detected Si is, of course, attributed to glass used as pixel substrate. The C peak in EDX spectra is due to some C cross-contamination during the sample handling process. The EDX spectra includes two non-labelled visible peaks. The first of them, located at 2.3 keV, is attributed to Au used as charge mitigation element for SEM analysis. The other one, located at 1.48 keV, is attributed to the Al sample-holder. 

The entire acquired EDX spectra are software processed (TEAM software, AMETEK-EDAX Inc., Mahwah, NJ, USA) utilizing the eZAF algorithm for standardless quantitative analysis of Pd, Sn and O elements in the transferred pixels 1–4. The results obtained are shown in Table 2. In the standardless quantitative process of element determination, all identified compositions are taken into account, but only the elements of interest are quantified (O, Pd and Sn) by resuming them to 100.

The as computed results highlights that the Pd content is gradually increasing from pixel 1 to pixel 4.

**Figure 6:**
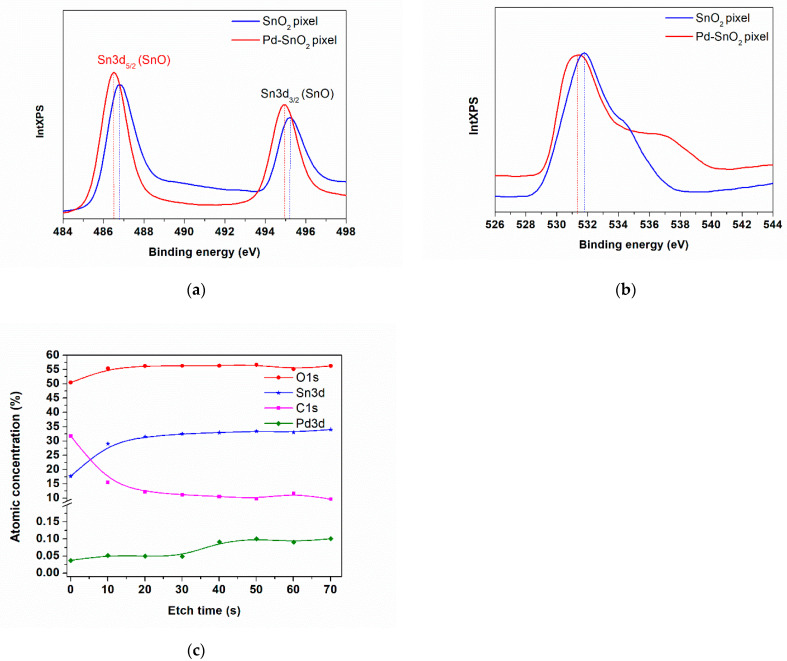
(**a**) Sn 3d, (**b**) O1s core-level XPS spectra of SnO_2_ and Pd-SnO_2_ pixels transferred at 100 mJ/cm^2^; (**c**) Evolution of O, C, Sn and Pd as a function of etching time.

As shown in Table 2, while the quantification error of O, an Sn element, is in the range of the standardless method, i.e., 3–10% [40], the quantification error of the Pd element is noticeably higher for pixel 1 and pixel 2. The origin of this increased error is, of course, related to the pixel 1 & 2 surfaces’ roughness, as revealed through AFM and SEM analysis. Further on, XPS is used to analyze the chemical composition and the nature of the chemical bonds of both the SnO_2_ and Pd-SnO_2_ pixel surfaces. The XPS spectra of the SnO_2_ and Pd-SnO_2_ pixels together with the atomic concentrations (shown in Table 3) of the SnO_2_ pixels transferred by LIFT at 100 mJ/cm^2^ laser fluence is shown in Figure 6a–c and Table 3, and is consistent with those reported elsewhere [41,42,43]. Spin–orbit doublet peaks at ≃486 eV (Sn^2+^ 3d_5/2_) and ≃495 eV (Sn^2+^ 3d_3/2_) can be observed in the Sn 3d spectra. As can be observed from Figure 6, the binding energies of both the Sn 3d_5/2_ and Sn 3d_3/2_ peaks for the SnO_2_ and Pd-SnO_2_ pixels have different values (i.e., 486.1 eV and 495.22 eV for SnO_2_ and 486.49 eV and 494.97 eV for Pd-SnO_2_). The trend of decreasing the binding energies of Sn 3d_5/2_ (by −0.3 eV in our case) by doping Pd in SnO_2_ has been reported earlier [43]. Although the mechanism has not been completely elucidated, the decrease in the Fermi level of the SnO_2_ is attributed to the role of electronic sensitizer of the Pd. The same observation can be made for the O1s peak of SnO_2_ as compared to the O1s peak of the Pd-SnO_2_ pixel (i.e., 531.74 eV for SnO_2_ and 531.3 eV for Pd-SnO_2_). The O1s peak of both SnO_2_ and Pd-SnO_2_ pixels is asymmetric and exhibits an evident shoulder in the higher binding energy part, as shown in Figure 6b, which could be attributed to the oxygen atoms chemisorbed at the surface [42]. Furthermore, due to the fact that no Pd peak could be found at the surface of the Pd-SnO_2_ pixels, we investigated the evolution of the O, C, Sn and Pd as a function of etching time, and our results are consistent with those from EDX, where an amount lower than 0.5% of Pd is present in the transferred pixels. 

**Figure 7:**
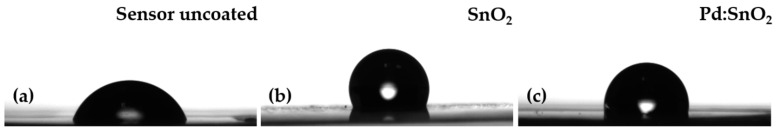
Contact angle images for (**a**) the uncoated working electrode, (**b**) the working electrode coated with a SnO_2_ pixel and (**c**) the working electrode coated with a Pd-SnO_2_ pixel.

Contact angle (CA) measurements are carried out to understand the surface wetting properties of the sensing electrode. The images of the water droplets on the uncoated, SnO_2_ and Pd-SnO_2_ coated sensors are shown in Figure 7. 

The contact angle of the uncoated sensor is 69.75° ± 0.69°, whilst the contact angle for the SnO_2_ coated sensor is 101.77° ± 0.24° and 95.4° ± 1.21° for Pd-SnO_2,_ respectively. The increase of the CA shows that the surface is more hydrophobic which is advantageous for self-cleaning of the sensor. Significantly, it demonstrates that the rLIFT results in a different surface wetting property, which is advantageous for any adsorption-based sensor development. 

### 3.3. Assessment of the LIFT Modified Electrodes Functionality

**Figure 8:**
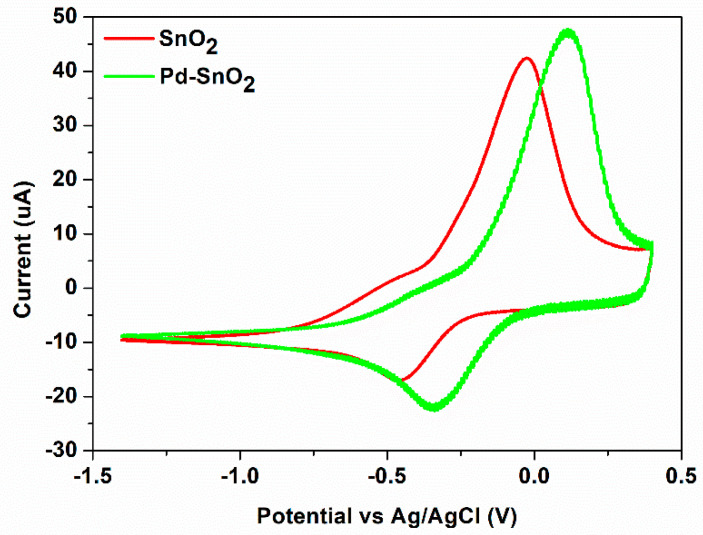
Cyclic voltammograms of Cu^2+^ (100 ppm) in 0.1 M acetate buffer solution (pH = 5.0) recorded on red SPE-based SnO_2_ and green SPE-based Pd-SnO_2_.

A typical cyclic volammogram of 100 ppm Cu^2+^ in 0.1 M acetate buffer solution (pH = 5.0 upH) obtained by using modified flexible screen-printed electrodes (SPE) based on PEDOT as working electrode is presented in Figure 8, the resulting data being presented in Table 4.

A single cathodic peak, I_pc_ is obtained during the forward scan at in the range of −476 mV → −330 mV, the Cu^2+^ being reduced to Cu^0^ by a two-electron transfer reaction (Cu^2+^ + 2e^−^ → Cu^0^). This is in good agreement with the study reported by Shaikh et. al., where they have demonstrated that bivalent copper is directly reduced to metallic copper in electrolyte solutions having pH > 4.08 [44]. In addition, the oxidation of copper (Cu^0^ → Cu^2+^ + 2e^−^) is observed during the reverse, with an anodic peak at around +45 mV. 

As can be observed in Table 4 the addition of SnO_2_ to the commercial SPE leads to a small decrease of both anodic and cathodic peaks current. The peak potential separation (∆E_p_) decreases to 429 mV, the sample showing a negative value of −31 mV for the anodic peak potential. The ratio between the current peaks is also lower (2.90) compared to the obtained value for the bare SPE. The addition of Pd nanoparticles to the SnO_2_-SPE sensor leads to an increase of the currents, especially for the cathodic peak. This reveals that the addition of Pd to SnO_2_ may provide the required conduction pathways at the surface of the electrode and ensures better electrochemical behavior. The peak potential separation is the same as that obtained for the commercial SPE and the current ratio is increased.

## 4. Conclusions

In this work, the combination of low-cost materials together with a facile and cost-efficient method to modify flexible electrodes for the detection of Cu ions has been evaluated. In particular, SnO_2_ and Pd-SnO_2_ thin film pixels were evaluated for their potential to amplify the signal of an electrochemical working electrode. Both the SnO_2_ and Pd-SnO_2_ thin film pixels were obtained by laser-induced forward transfer (LIFT) from metal acetylacetonates precursors which are decomposed under UV light. Extensive evaluation of the morphological and chemical properties of the transferred materials reveals that LIFT was successfully applied to obtain chemically stable SnO_2_ and Pd-SnO_2_ pixels which exhibit a high roughness. In addition, the contact angle measurements carried out onto the transferred SnO_2_ and Pd-SnO_2_ pixels highlight the possibility to tune the wettability of the screen-printed electrodes (SPE) by LIFT. 

Finally, compared to the bare screen-printed electrodes, the addition of Pd nanoparticles to the SnO_2_-SPE sensor, leads to an increase of the currents, especially for the cathodic peak. Thus, the LIFT fabricated Pd-SnO_2_ electrodes could be used as a classic sensor for Cu ions detection. This result also suggests that this simple method could be used for decoration and modification of electrodes for the detection of other toxic ions.

## Figures and Tables

**Table 1:** AFM parameters that describe the surface morphology for (40 µm × 40 µm) scanned areas.

Donor	Min (nm)	Max (nm)	Rq (nm)	Rsk	Thickness (nm)
SnCl_2_(acac)_2_	−457.323	781.542	156.176	−1.105	1085
Pd-SnCl_2_(acac)_2_	−519.386	781.174	97.251	−0.379	895

**Table 2:** EDX analysis using the standardless ZAF quantification method of Pd, Sn and O elements in the transferred pixels 1–4.

	Pixel 1			Pixel 2			Pixel 3			Pixel 4		
Element	wt %	Atomic %	Error %	wt %	Atomic %	Error %	wt %	Atomic %	Error %	wt %	Atomic %	Error %
**OK**	51.34	88.67	4.29	46.92	88.77	4.52	40.09	83.23	4.82	41.88	84.23	4.89
**PdL**	0.04	0.01	17.45	0.07	0.02	15.91	0.26	0.08	10.00	0.39	0.12	11.87
**SnL**	48.62	11.32	3.04	53.02	13.22	2.89	59.65	16.69	2.67	57.73	15.65	2.68

**Table 3:** Atomic concentrations (%) of SnO_2_ pixels transferred by LIFT at 100 mJ/cm^2^.

Sn 3d_5_	O1s	C1s
20.65 ± 2.5	66.3 ± 3.5	13.05 ± 4

**Table 4:** Data obtained from voltammograms recorded for Cu^2+^ in acetate buffer on different SnO_2_ and Pd-SnO_2_ based sensors.

Sensor	Cu^2+^ Concentration	pH	Peak Current (μA)		Peak Potential (mV)		∆Ep = Epa − Epc	|ipa/ipc|	Ref.
			(−) ipc	ipa	(−) Epc	Epa			
Commercial SPE-PEDOT	100 ppm	5	11.41	42.9	430	26	450	3.76	-
GCE	ca. 188 ppm	1.03	50.99	140.24	82	53	135	2.75	[44]
GCE	ca. 135 ppm	5.3	125	148.2	301.4	291.52	592.92	1.19	[45]
Modified-SPCE	1 ppm	8	-	<2	-	-	-	-	[46]
SnO_2_	100 ppm	5	10.01	38.03	460	−31	429	2.90	This work
Pd-SnO_2_	100 ppm	5	15.18	44.06	340	110	450	3.79	This work

GCE—glassy carbon electrode; SPCE—screen-printed carbon electrode.

## Data Availability

The data used to support the findings of this study are available from the corresponding author upon request.
